# Genome-wide DNA methylome analysis reveals epigenetically dysregulated non-coding RNAs in human breast cancer

**DOI:** 10.1038/srep08790

**Published:** 2015-03-05

**Authors:** Yongsheng Li, Yunpeng Zhang, Shengli Li, Jianping Lu, Juan Chen, Yuan Wang, Yixue Li, Juan Xu, Xia Li

**Affiliations:** 1 College of Bioinformatics Science and Technology, Harbin Medical University, Harbin, China; 2 Shanghai Center for Bioinformation Technology, Shanghai, People's Republic of China

## Abstract

Despite growing appreciation of the importance of epigenetics in breast cancer, our understanding of epigenetic alterations of non-coding RNAs (ncRNAs) in breast cancer remains limited. Here, we explored the epigenetic patterns of ncRNAs in breast cancers using published sequencing-based methylome data, primarily focusing on the two most commonly studied ncRNA biotypes, long ncRNAs and miRNAs. We observed widely aberrant methylation in the promoters of ncRNAs, and this abnormal methylation was more frequent than that in protein-coding genes. Specifically, intergenic ncRNAs were observed to comprise a majority (51.45% of the lncRNAs and 51.57% of the miRNAs) of the aberrantly methylated ncRNA promoters. Moreover, we summarized five patterns of aberrant ncRNA promoter methylation in the context of genomic CpG islands (CGIs), in which aberrant methylation occurred not only on CGIs, but also in regions flanking CGI and in CGI-lacking promoters. Integration with transcriptional datasets enabled us to determine that the ncRNA promoter methylation events were associated with transcriptional changes. Furthermore, a panel of ncRNAs were identified as biomarkers that discriminated between disease phenotypes. Finally, the potential functions of aberrantly methylated ncRNAs were predicted, suggestiong that ncRNAs and coding genes cooperatively mediate pathway dysregulation during the development and progression of breast cancer.

The development of human breast cancer is mediated by both genetic and epigenetic alterations of the cell^1^,^2^. Since the discovery of altered DNA methylation in human cancer, DNA methylation studies of breast cancer have used methodologies of varying scale, focusing on a few coding genes or regions assumed to be functionally important, such as promoters and CpG islands (CGIs)^3^,^4^. Although it is well understood that most of the mammalian genome is transcribed, producing non-coding RNAs (ncRNAs), the genome-wide methylation patterns of ncRNAs in breast cancer remain largely unknown.

NcRNA transcripts have been categorized into several groups based on their size, which is the most popular classification method. These classes include the well-annotated microRNAs (miRNAs) and long ncRNAs (lncRNAs). LncRNAs account for approximately 81.8% of all ncRNAs^5^. Although the molecular basis of the functions of many lncRNAs is just emerging, much evidence indicates that lncRNAs play intricate roles in the regulation of a wide variety of biological processes, such as imprinting and gene expression at the transcriptional level^6^,^7^,^8^. Considering the potential functions of lncRNAs, their transcription must be tightly regulated. Aberrant expression of lncRNAs has appeared in prevalent cancer types, including breast cancer. One notable example is HOTAIR, which is over-expressed in breast cancers; loss of HOTAIR reduces the invasiveness of breast cancer^9^. Another example is MIR31HG, which is expressed abundantly in non-invasive breast cancer cell lines of the luminal subtype^10^. Although lncRNAs have been demonstrated to participate in the modulation of gene expression^11^, the epigenetic regulation of lncRNAs remains poorly understood. Recent studies have described aberrant methylation of specific lncRNAs in breast cancers. However, studies of aberrant epigenetic regulation patterns in lncRNA genes at a global scale are scarce.

In addition, miRNAs are a recently discovered and well-characterized class of ncRNAs^12^. MiRNAs are important regulators of gene expression and are frequently dysregulated in cancer^13^,^14^; aberrant DNA methylation is an epigenetic mechanism that is involved in the process of miRNA dysregulation^15^,^16^,^17^. Aberrant DNA methylation events associated with the silencing of individual miRNAs have been demonstrated in many cancer types, including breast cancer^18^,^19^. Some of these miRNAs function as tumor suppressors (such as miR-203, miR-195 and miR-497) and the down-regulation of these miRNAs due to aberrant hypermethylation is associated with increased malignancy or metastatic potential in breast cancer^20^,^21^. Using 5-methylcytosine immunoprecipitation coupled to miRNA tiling microarray hybridization, Vrba et al. have demonstrated that miRNA gene promoters are frequent targets of aberrant DNA methylation in human breast cancer^22^, indicating an important role of DNA methylation in miRNA dysregulation in cancer. However, only 167 miRNAs were analyzed in their study, accounting for only 10% of all miRNAs in the genome. To our knowledge, the comprehensive analysis of the methylation of miRNA genes in breast cancer has yet to be performed.

Next-generation sequencing technologies have emerged as powerful tools that enable whole-genome profiling of epigenetic modifications, including DNA methylation. For instance, the MBDCap-seq protocol, is a technique used to identify methylated DNAs using a methyl-CpG binding domain (MBD) protein column followed by next-generation sequencing. The low cost and unbiased generation of the methylation profiles of both coding and non-coding regions render this technique as suitable for genome-wide methylation profile analysis. The Cancer Methylome System (CMS)^23^ has recently used high-throughput sequencing technology to generate DNA methylation profiles in a cohort of 87 breast samples (77 cancer samples and 10 normal control samples). This study was a comparative analysis of the methylomes generated by the previous unbiased systematic effort to determine the aberrant methylation patterns of ncRNAs, and to provid the precise genomic locations that undergo methylation changes. The data used in this study represent a highly valuable public resource understanding the epigenetic regulation of the breast cancer genome and for identifying ncRNAs as therapeutic targets.

## Results

### Global differences in DNA methylation between breast cancer and normal control samples

We performed comprehensive comparative analyses of the DNA methylation profiles of normal control and breast cancer samples, which were downloaded from the CMS. First, by computing the average intensity correlation of methylation across all breast cancer and normal control samples, we found that the control and breast cancer samples were classified into two different groups (Figure S1A). Moreover, the variation of the correlation strength among the breast cancer group was higher than that of the normal control group (Figure S1B), suggesting that the methylation profiles among individuls with breast cancer may display some heterogeneity.

Next, the differentially methylated regions (DMRs) across the entire genome were identified. Approximately 838,817 hypo-methylated DMRs were identified in the breast cancer samples, and this result was approximately 16-fold greater than that of hyper-methylated DMRs (Figure S1C). By investigating the genomic distribution of the DMRs, we found that the hyper- and/or hypo-methylated DMRs were dispersed throughout multiple chromosomes, and their distributions were similar (Figure S1D). In summary, the breast cancer samples contained extensive methylome alterations.

### Aberrant methylation is associated with ncRNAs in breast cancer

**Figure 1:**
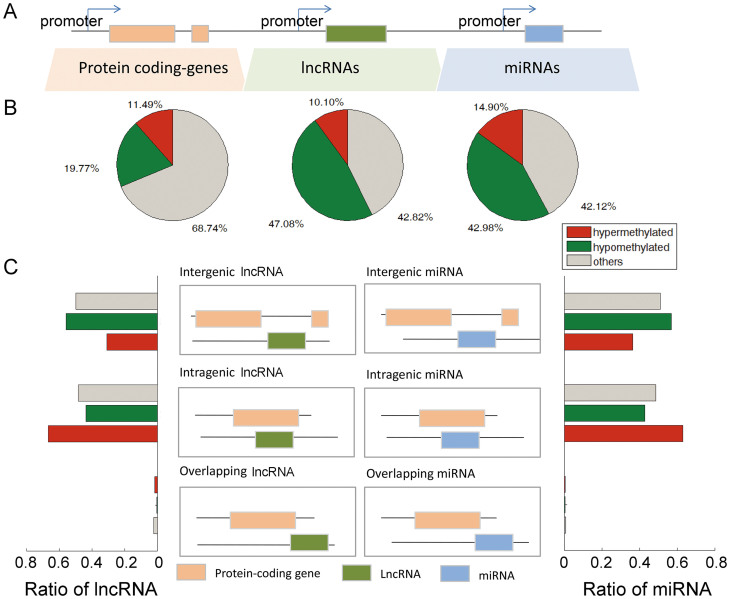
Characterization of the genome-wide methylation patterns of ncRNAs. (A) Schematic representing the analyzed genomic features. (B) Pie charts representing the percentage of coding and non-coding promoters that overlapped with hyper- and hypo-methyalted DMRs in breast cancer. The green pie charts represent the percentage of the regopms covered by hypo-methylated DMRs, and the red pie charts represent the percentage of the regions covered by hypermethyalted DMRs. (C) LncRNAs and miRNAs were classified into different subcategories based on their location relative to protein-coding genes. The bar graphs show that the majority of intragenic ncRNAs were overrepresented with respect to hypermethylation and that most intergenic ncRNAs were hypomethylated in the breast cancer samples (all p-values < 0.05, Fisher's exact test).

NcRNAs (including miRNAs and lncRNAs) are important regulators of gene expression; however, the genome-wide aberrant methylation of ncRNAs in breast cancer has been poorly understood. By mapping the DMRs to the ncRNA and protein-coding genes, we observed wide methylation changes in the promoters of ncRNA genes, which were more frequent than those of protein-coding genes (Figure 1A and 1B). Specifically, approximately 57.18% of the lncRNA promoters and 57.88% of the miRNA promoters were aberrantly methylated. By contrast, only 31.26% of the promoters of coding genes were aberrantly methylated in breast cancer, some of which were in known disease-related genes, such as HOXB2, FGF4 and TTK. By further investigating the direction of methylation dsyregulation, we identified 1,359/278 hypermethylated lncRNA/miRNAs and 6,336/802 hypomethylated lncRNA/miRNAs (Table S1). Among these identified miRNAs, many have been demonstrated to be involved in the process of tumorigenesis, such as hsa-mir-29b^16^, hsa-mir-21^24^ and hsa-mir-10b^25^.

Previous reports have suggested that different biotypes of ncRNAs perform distinct functions^26^,^27^. Thus, these ncRNAs were further classified into three biotypes based on their location with respect to protein-coding genes: intergenic, intragenic and overlapping ncRNAs. Intergenic lncRNAs comprised the majority of the aberrantly methylated ncRNA promoters (51.45% of all lncRNAs and 51.57% of all miRNAs) in the breast cancer samples (Figure 1C). Most of these intergenic lncRNAs had not previously been detected via microarray analysis, and our genome-wide analysis produced new candidates for further functional analysis. Specifically, approximately 31.79% of the hypermethylated lncRNAs and 55.84% of the hypomethylated lncRNAs were intergenic. Similar to the dysregulation patterns of lncRNAs, 36.33% of the hypermethylated miRNAs were intergenic (Figure 1C). Intragenic ncRNAs comprised another interesting type of ncRNAs. We found that most intragenic ncRNAs shared the same -or largely overlapping promoters with their host genes. Moreover, for the ncRNA-host promoter pairs displaying an overlap of less than 50%, 32.65% and 24.79% of the lncRNAs and miRNAs, respectively, exhibited the same directional change in methylation as their host genes. These results indicate that most intragenic ncRNAs tend to display identical methylation patterns to their host genes. However, it cannot be ignored that several ncRNAs display independent methylation patterns.

### Aberrant epigenetic regulation patterns of ncRNA promoters in the context of CGIs

CGIs have been found to be functionally important in cancer based on DNA methylation studies. To determine whether CGIs tend to be aberrantly methylated in breast cancer, the DMRs were mapped to CGIs. As a result, we observed that 31.85% of the CGIs were hyper-methylated in the breast cancer samples (Figure S2A). In addition to CGIs, CGI shores have been demonstrated to be frequently aberrantly methylated in colon cancer^16^. However, the investigation of the methylation patterns of CGI shores in breast cancer has been hampered by a lack of genome-wide profiling. Here, we found that approximately 40% of CGI shores were aberrant methylated and the number of 5' shores and 3' shores displaying hyper- or hypo-methylation appeared to be equal (Figure S2A). Moreover, we compared the methylation frequency distributions of hypo-methylated CGIs with those of hyper- methylated CGIs and observed opposing trends (Figure S2B and S2C). The hyper-methylation frequency was extremely high on CGIs, whereas the hypomethylation frequency was substantially high on CGI shores. Specifically, for 88.69% of the hypermethylated CGIs, their corresponding 5′ or 3′ shore regions were also hyper-methylated (Figure S2D). By contrast, the hypo-methylated DMRs tended to reside on CGI shores but not on CGIs, and the 5′ and 3′-CGI shores were rarely simultaneously hypomethylated (Figure S2E).

**Figure 2:**
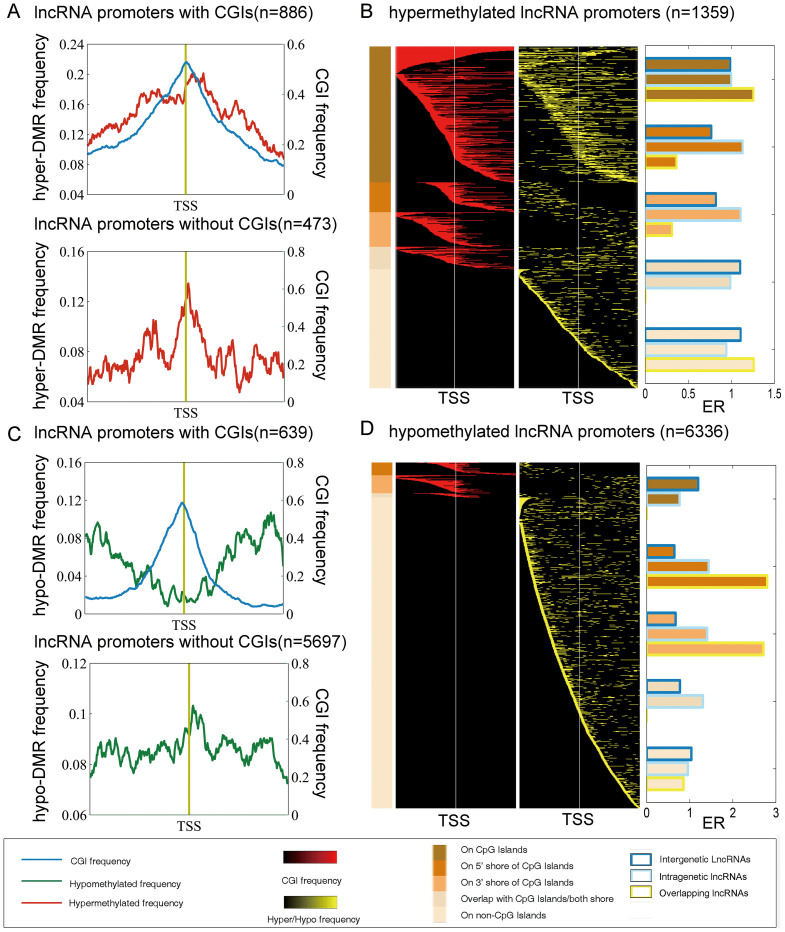
Aberrant methylation patterns surrounding the TSSs of the lncRNAs in breast cancer. (A) Average aberrant hypermethylation frequency of 886 lncRNA promoters containing CGIs and 473 lncRNA promoters lacking a CGI. (B) Heat map of the CGI frequency and the aberrant hypermethylation frequency in breast cancer. A total of 1359 lncRNA promoters contained a hypermethylated region (yellow) were identified. Each row represents a unique promoter region at a 10-bp window size, covering the +/−2-kb region flanking the transcription start sites. The location of a CGI (red) in the aberrantly methylated lncRNA promoters is shown in the first column. The promoters are ordered according to the location of methylation on a CGI, or adjacent to the CGI (CGI shore); the promoters that lacked a CGI are represented with different shades of brown on the left. ER, enrichment ratio. (C) Average aberrant hypomethylation frequency of 639 lncRNA promoters containing CGIs and 5697 lncRNAs lacking a CGI. (D) Heat map of the CGI frequency and the aberrant hypomethylation frequency in breast cancer. The ratio the of lncRNA subtypes for each pattern is shown adjacent to the heat map.

Recently, Weber et al. reported that gene promoters containing weak CGIs unbound to RNA polymerase II were frequently methylated^28^. Thus, the relevance of specific characteristics of DNA methylation, such as the location of aberrant methylation, or the association between CGI and CGI shore methylation, to the development of cancer merits investigation. Next, we examined the aberrant methylation patterns of ncRNAs in the context of CGIs and identified several distinct aberrant methylation patterns on lncRNA promoters. Overall, 34.81% (n = 473) of the hypermethylated lncRNA promoters lacked a CGI and displayed aberrant hypermethylation arounding the transcription start site (TSS) (Figure 2A). The remaining 65.19% (n = 886) of the hypermethylated lncRNA promoters contained CGIs, and among these promoters, four distinct aberrant methylation patterns were classified (Figure 2B): (1) aberrant methylation predominantly confined to CGIs; (2) aberrant methylation located on the 5′ shore of the CGIs; (3) aberrant methylation located on the 3′ shore of the CGIs and (4) aberrant methylation that overlapped with the CGI shores or an overlap of less than 50% between the CGIs and the DMR. We found that aberrant hypermethylation was predominantly confined to CGIs or their 5′ and 3′ shores (Figure S3A and Table S2). Next, we calculated the average aberrant methylation frequency by dividing the promoters into 10 bp fragments and observed that the aberrant hypermethylation frequency was particularly high immediately downstream of the TSS in these hypermethylated promoters (Figure 2A). This region has generally been regarded as the core promoter^29^, in which RNA polymerase II is recruited to the DNA and initiates transcription. Aberrant methylation in core promoter regions may lead to transcriptional inactivation/activation of cancer-related genes and plays an integral role in tumorigenesis. For the hypomethylated lncRNA promoters in the breast cancer samples, we found that nearly 89.91% of the hypomethylated lncRNA promoters lacked a CGI (Figure 2C and Figure S3A); the aberrant hypomethylation frequency peak was immediately downstream of the TSS (Figure 2C), similar to that for aberrant hypermethylation. However, the aberrant methylation frequency of the hypomethylated promoters containing CGIs was distinct from that of other promoters because these promoters displayed the lowest aberrant hypomethylation frequency surrounding the TSS (Figure 2C). Moreover, we found that intragenic lncRNAs comprised the majority of each pattern of aberrantly methylated lncRNA promoters. In addition, the intergenic lncRNAs were overrepresented among the hypomethylated lncRNAs (Figure 2B and 2D).

**Figure 3:**
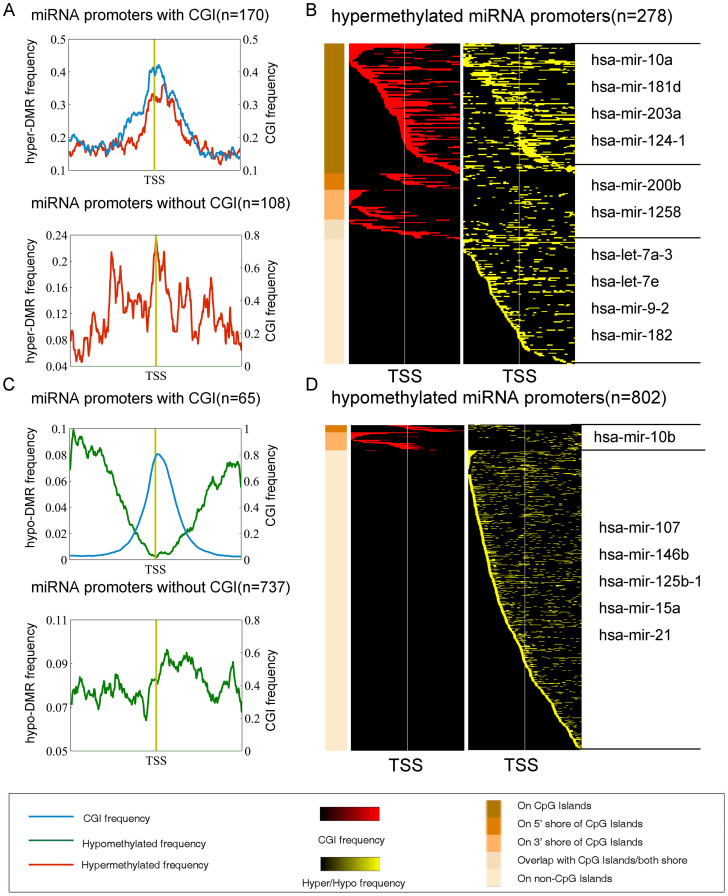
Aberrant methylation patterns surrounding the TSSs of miRNAs in the breast cancer samples. (A) Average aberrant hypermethylation frequency of 170 promoters containing CGIs and that of 108 promoters that lacked a CGI. (B) Heat map of the CGI frequency and the aberrant hypermethylation frequency in breast cancer. Representative miRNAs in each group are shown on the right. (C) Average aberrant hypomethylation frequency of 65 promoters containing CGIs and that of 737 promoters that lacked a CGI. (D) Heat map of the CGI frequency and the aberrant hypomethylation frequency in breast cancer. Representative miRNAs in each group are shown on the right.

We also found that the global distribution of the aberrant methylation frequency of miRNAs was similar to that of lncRNAs (Figure 3 and Table S2). Specifically, the aberrantly hyper-methylated miRNA promoters were confined to CGIs, and the hypo-methylated miRNA promoters lacked CGIs (Figure 3B). Moreover, the aberrant methylation frequency, especially the hypermethylation frequency, was increased surrounding the TSS, implying that miRNA promoters were frequent targets of aberrant DNA methylation in human breast cancer^22^. These results suggest a similar epigenetic mechanism between different types of ncRNAs.

### Regulation of ncRNA expression by DNA methylation

**Figure 4:**
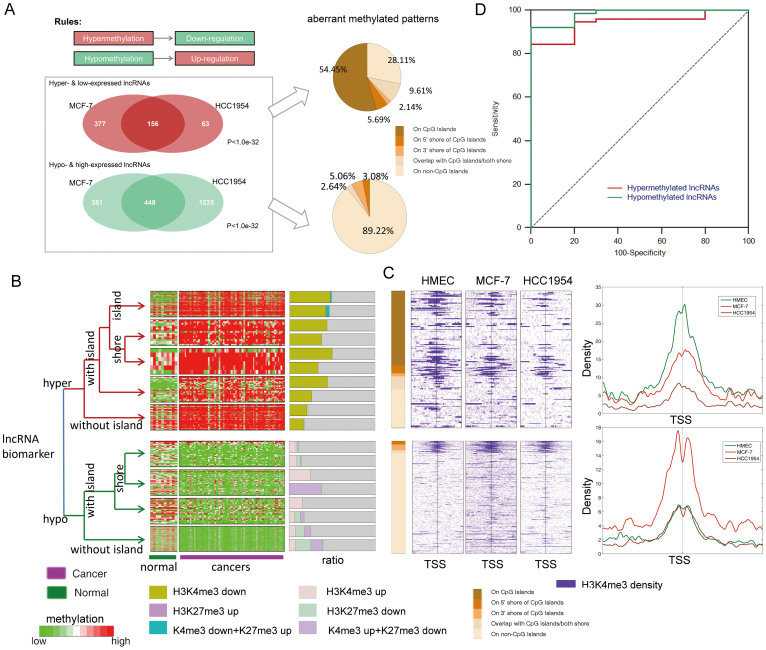
Regulation of lncRNA expression by DNA methylation in breast cancer. (A) The aberrantly methylated and expressed lncRNAs in the two cell lines significantly overlapped. (B) Clustering map of selected lncRNA biomarkers for CMS methylation. The lncRNAs were grouped based on their aberrant methylation patterns. The bar graph adjacent to the heat map shows the percentage of lncRNAs in that group covered by an aberrant histone modification. (C) Colored profiles of the H3K4me3 sequencing read densities from the HMEC, MCF-7 and HCC1954 cell lines. Each profile shows the 4-kb regions surrounding the aberrantly methylated lncRNAs. The average profiles are shown on the right of the colored profiles. (D) ROC map of the selected markers.

To explore the functional consequences of aberrant methylation, we next examined the association between ncRNA methylation events and transcriptional changes. Based on the repressive effects of DNA methylation, two scenarios of particular biological relevance were considered. In the first scenario, reduced expression of ncRNA is due to hypermethylation (silencing), and in the second scenario, increased expression of ncRNA is due to hypomethylation (activating). As a result, 156 and 448 candidate silencing and activating lncRNAs candidates, respectively, were identified in both cell lines (Figure 4A), which were distributed in the patterns described above. Moreover, consistent with previous studies of coding genes, the aberrantly hypermethylated lncRNAs containing CGIs were more likely to be associated with transcriptional repression. Approximately 54.45% of the hypermethylated lncRNAs containing CGIs were associated with transcriptional repression (Figure 4A). As both DNA methylation and histone modification are involved in establishing patterns of gene expression during the progression of cancer, we next explored whether aberrant methylation of lncRNAs was associated with altered histone modification (Text S1). Notably, approximately 50% of the hypermethylated lncRNAs displayed reduced H3K4me3 or elevated H3K27me3 levels, whereas, approximately 30% of the hypomethylated lncRNAs were associated with elevated H3K4me3 or reduced H3K27me3 levels (Figure 4B). Moreover, the aberrant DNA hypermethylation of ncRNAs was more likely to coincide with aberrant modification of H3K4me3 than that of H3K27me3. By contrast, ncRNA hypomethylation was associated with an alteration of the H3K27me3 levels. This result is consistent with previous observations that H3K27me3 and DNA methylation are mutually exclusive, especially on CGIs^30^. Specifically, we found that the incidence of histone modification changes was extremely high at the TSS, reinforcing the notion that aberrant epigenetic modifications were strongly biased toward the core promoter regions (Figure 4C).

The ncRNA or protein-coding genes display aberrant expression might serve as biomarkers for cancer diagnosis. Therefore, we examined whether these lncRNAs served as potential biomarkers for early diagnosis. The potential biomarkers that were examined consisted of the activating and silencing lncRNAs identified above. The results revealed that these activating and silencing lncRNAs distinguished breast cancer samples from healthy control samples, which might aid in the future development of diagnostic biomarkers for breast cancer. The diagnostic value of these lncRNAs was evaluated based on area under the receiver operation characteristic (ROC) curve (AUC) analysis. The ROC curves of the hyper- and hypomethylated lncRNAs displayed AUC values of 0.944 and 0.983, respectively, using the CMS dataset (Figure 4D).

**Figure 5:**
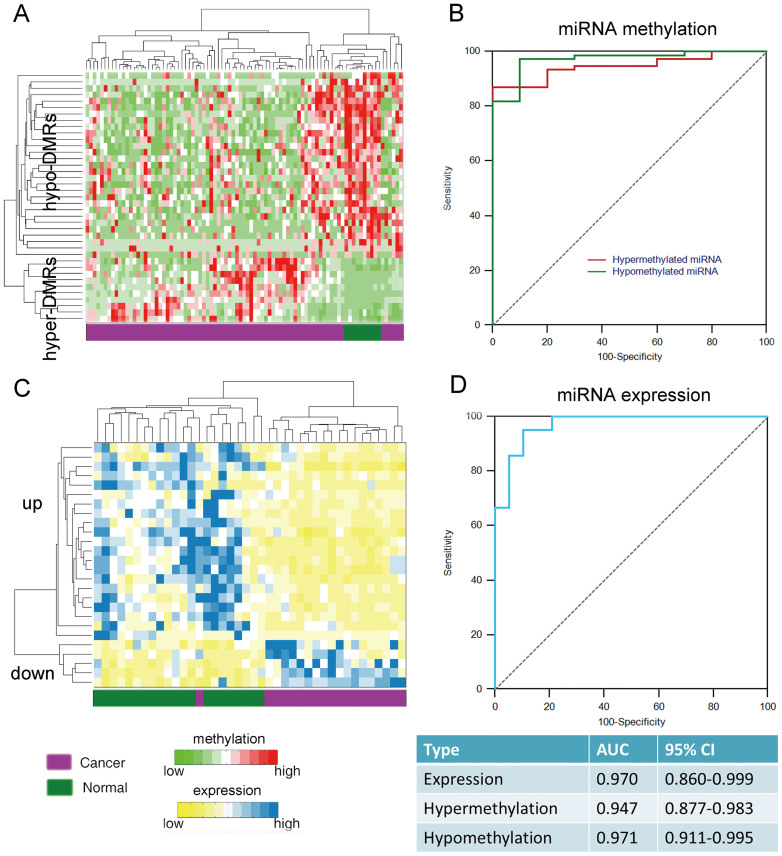
Regulation of miRNA expression by DNA methylation in breast cancer. (A) and (C) Clustering map of the selected miRNA biomarkers in the CMS methylation dataset and TCGA expression dataset, respectively. (B) and (D) ROC analysis of these miRNA biomarkers.

Furthermore, miRNA biomarkers were identified, including 26 (5 hypermethylated and 21 hypomethylated) miRNAs. Three of these five hypermethylated miRNAs, were confined to CGIs. Most of the hypomethylated miRNAs lacked a CGI in their promoters; aberrant methylation was located on the CGI shores of several miRNA promoters, such as hsa-mir-106a, hsa-mir-1292 and hsa-mir-3613. Based on hierarchical clustering analysis of the samples according to the methylation and expression of these 26 miRNAs (Figure 5A and 5C), most breast cancer tissues were distinguishable from adjacent benign tissues, with overall AUCs larger than 0.90 in the CMS and TCGA datasets (Figure 5B and 5D). Notably, most of these miRNAs were previously associated with breast cancer (Table S3).

### Epigenetically dysregulated ncRNAs disrupt functions associated with breast cancer

**Figure 6:**
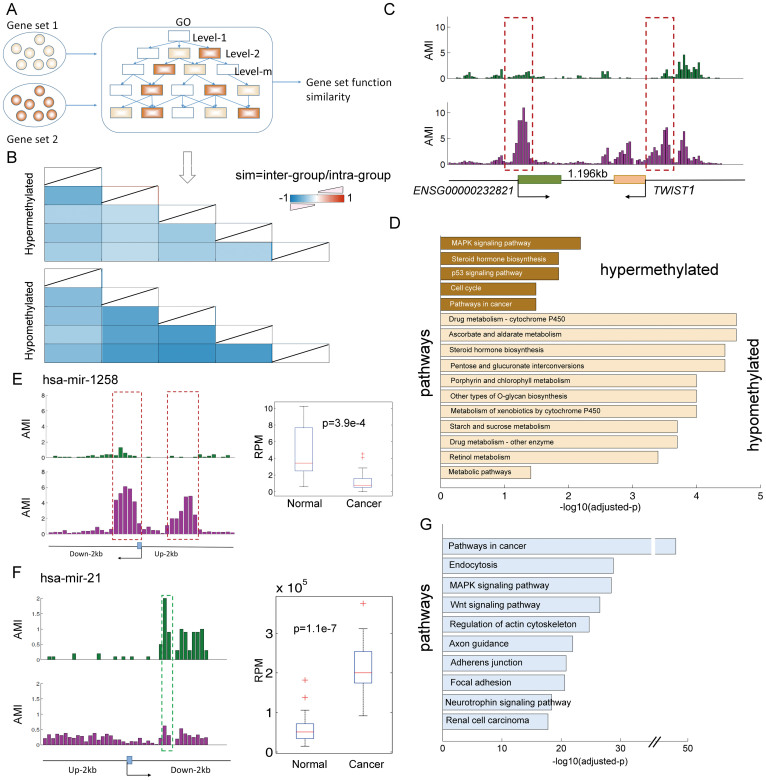
Aberrantly methylated ncRNAs widely disturb functions associated with breast cancer. (A) The system used to calculate the gene set functional similarity. (B) The genes that displayed similar aberrant methylation patterns exhibited with high functional similarity. The similarity score was normalized to the intra-classes. (C) Representative lncRNA and protein-coding genes. The average methylation intensities (AMIs) in the control and breast cancer samples are shown. Green, control samples; Pink, cancer samples. (D) The biological processes enriched by adjacent genes of lncRNA displaying similar aberrant methylation patterns. (E) The average methylation and expression levels of hsa-mir-1258 in the normal and breast cancer samples. RPM, reads per million. (F) The average methylation and expression levels of hsa-mir-21 in the control and breast cancer samples. (G) The top 10 pathways enriched by targets of miRNA biomarkers.

An increasing number of lncRNAs have been characterized; however, the functions of most lncRNA genes remain unknown. In addition, the functional prediction of lncRNAs is hampered by the lack of collateral information, such as molecular interaction data and expression profiles. The large-scale functions annotated to lncRNAs were primarily predicted based on co-expression and genomic adjacency^31^,^32^. However, despite similar expression patterns, groups of functionally related genes can be further distinguished at the chromatin level^33^. Thus, we hypothesized that genes displaying similar aberrant methylation patterns may perform similar functions. To test this hypothesis, we first investigated the aberrant methylation patterns of the protein-coding genes. We found that the protein-coding genes shared similar aberrant methylation patterns with the ncRNA genes (Figure S4). Next, we determined the functional similarity of the gene set between genes displaying distinct aberrant methylation patterns (Figure 6A and Text S1). Consistent with our hypothesis, we found that genes displaying similar aberrant methylation patterns exhibited greater functional similarity than genes displaying distinct methylation patterns (Figure 6B). In addition, the genes that display the same directional change in methylation exhibited greater functional similarity than those that displayed oppositing directional changes in methylation (Figure S5).

As the general large-scale functional annotation of lncRNAs has been based on the ‘guilt-by-association’ principle, we applied this principle and aberrant methylation patterns to predict the probable functions of the aberrantly methylated and expressed lncRNAs identified above. For example, the lncRNA ENSG00000232821 displayed hypermethylation confined to its CGI. We found that the coding gene -TWIST1, which is adjacent to this lncRNA, also displayed hypermethylation confined to its CGI (Figure 6C). TWIST1 is an important transcription factor that has been implicated in cell lineage determination and differentiation. TWIST1 promoter methylation has been demonstrated to be significantly more prevalent in malignant than in healthy breast tissue. It is reasonable to infer that this lncRNA may also be involved in development of breast cancer. We found that the potential functions of this lncRNA based on the NONCODE database were the regulation of growth and development, suggesting that this lncRNA plays important roles in the development of cancers. Next, we aimed to explore the dysregulated pathways corresponding to different patterns of aberrantly methylated lncRNA biomarkers. We found that only the hypermethylated lncRNAs confined to CGIs and the hypomethylated lncRNAs lacking CGIs were enriched in pathways directly associated with the development and progression of breast cancers (Figure 6D and Table S4). The hypermethylated lncRNAs primarily affected the cell cycle and signaling pathways, whereas the hypomethylated lncRNAs predominantly disturbed most of metabolic pathways (Table S4). Cancer cells require metabolism to sustain their existence, including the support of several functions, such as cell maintenance, proliferation and motility. Common to all of these activities is the demand for energy^34^. These results indicated that lncRNA hypomethylation may be associated with carcinogenesis by dysregulation of the signaling and metabolic pathways.

It is well understood that miRNAs perform their important functions via targets; functional enrichment analysis revealed that the predicted targets of these 26 miRNA biomarkers, were implicated in functions that play direct and important roles in tumor growth and metastasis (Figure 6G and Table S5). For example, hsa-miR-29b was highly over-expressed in breast cancer, and directly enhancing the hsa-miR-29b mediated impairment of apoptosis and increasing tumor cell migration and invasion by targeting the tumor suppressor PTEN^35^. In the present study, we found that although the promoter of miR-29b lacked a CGI, it was hypomethylated, providing an epigenetic perspective of the aberrant expression of hsa-miR-29b in cancers. Another example is the tumor suppressor- hsa-miR-1258, which was hypermethylated on the 3′ CGI shore and was under-expressed in breast cancer samples in the present study (Figure 6E). Heparanase (HPSE) is a potent pro-tumorigenic, pro-angiogenic, and pro-metastatic enzyme that is over-expressed in brain metastatic breast cancer (BMBC). Studies have shown that hsa-miR-1258 inhibits the expression and activity of HSPE in BMBC cells, and stable expression of hsa-miR-1258 in BMBC cells inhibits HSPE-induced cell invasion in vitro and brain metastasis in an experimental model^36^. In addition, high hsa-miR-21 expression was associated with mastectomy, larger tumor size, higher tumor stage, higher tumor grade, estrogen receptor status. Increasing studies have identified many downstream targets of hsa-miR-21, such as WNT5A, PTEN and BRCA1, in various types of cancers^24^. Consistent with previous studies, hsa-miR-21 was significantly highly expressed in our analyzed breast cancer samples (p = 1.1e–7, t test). However, the mechanism underlying the high expression of hsa-miR-21 remains largely unknown. We found that the promoter of this miRNA lacks a CGI and is hypomethylated in breast cancer samples (Figure 6F), suggesting a possible epigenetic role in the regulation of its activity in breast cancer. Moreover, we found that some of the targets of this miRNA, such as BRCA1, CDK6 and WNT5A, were hypermethylated. Mutation of the tumor suppressor gene BRCA1 is an important contributor to the hereditary breast cancer; however, BRCA1 mutations have not been detected in certain types of breast cancer, suggesting that DNA methylation and/or miRNA-mediated repression of BRCA1 may participate in the development of breast cancers^37^. Our analysis indicated that hypomethylation of miRNA regulators and hypermethylation of the BRAC1 gene may be another repression mechanism of this tumor suppressor gene.

### NcRNAs and coding genes coordinately mediate pathway dysregulation in breast cancer

**Figure 7:**
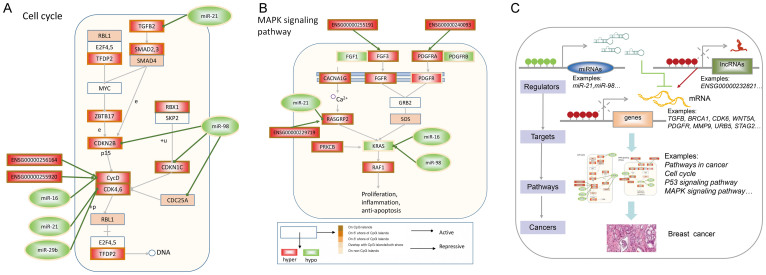
NcRNAs and coding genes cooperatively mediated pathway dysregulation in breast cancer. (A) The cell cycle pathway was dysregulated by ncRNAs and coding genes. Only the representative component of this pathway is shown. The hypermthylated genes are marked in red, and the hypomethylated genes are marked in green. The aberrant methylation patterns are labeled on the edge of the rectangle. The experimentally validated miRNA-mediated regulations from TarBase and adjacent lncRNAs with common aberrant methylation patterns were linked. (B) The MAPK signalling pathway. Only the representative component of this pathway is shown. (C) A model illustrating the dysregulated cellular network intertwined by ncRNAs and coding genes, ncRNA and coding genes cooperatively mediate the pathway dysregulation.

Tumorigenesis is a complex dynamic biological process that involves multiple steps of genetic and epigenetic alterations^38^. However, our understanding of this complex network intertwined by coding genes and ncRNAs in cancer biology remains at an early stage. We found that aberrant methylation of either lncRNA or miRNA can perturb specific common pathways, such as the cell cycle, and MAPK signaling pathways, indicating that ncRNAs mediate the dysregulation of these pathways in a coordinated manner. As one of the most important cellular processes, the cell cycle is precisely regulated in all organisms. Dysregulation of the cell cycle can lead to catastrophic cellular events, e.g., premature apoptosis or abnormal proliferation, which are causes of cancers^39^. We found that the lncRNA and miRNA regulated key components of this pathway. One such example is the CDK6 gene, which is responsible for modulating the activities of growth-suppressing Rb family proteins. Evidence has shown that normal human mammary epithelial cells (HMECs) exhibit a high level of CDK6 activity, but all breast tumor-derived cell lines exhibit lower CDK6 activity levels, with several having little or no CDK6 activity^40^. Our analyses demonstrated that DNA methylation may be one mechanism by which these cell cycle-associated genes are repressed (Figure 7A). Moreover, we found that this gene was targeted by two hypomethylated miRNAs, hsa-miR-21 and hsa-miR-29b. The hypomethylation of these two key regulators may further repress the activity of CDK6, implying complementary effects of DNA methylation- and miRNA-mediated regulation. Another example is the hypermethylated gene- CCND2 (Figure 7A), which was regulated by a hypomethylated miRNA (hsa-miR-16). This cyclin forms a complex with and functions as a regulatory subunit of CDK4 or CDK6, the activity of which is required for the G1/S transition in the cell cycle^41^. In addition, two hypermethylated lncRNAs share a similar aberrant methylation pattern to this gene (hypermethylated on CGIs), implying that these two lncRNAs play key roles in cell cycle regulation.

Another dysregulated pathway is the MAPK signaling pathway, which controls fundamental cellular processes, such as growth, proliferation, differentiation, migration and apoptosis^42^. It was revealed in our study that most genes in this pathway were aberrantly methylated in breast cancer (Figure 7B). Some of these genes, such as FGFR1, KRAS and PRKCB, were reported to be involved in tumorigenesis. Correlations between the expression of FGFRs and breast cancer progression have been observed^43^, and our study indicated that DNA hypomethylation may be one mechanism responsible for the repression of FGFR genes in breast cancer. The Ras gene is another important gene in breast cancer and belongs to the Ras oncogene family, which has been very extensively studied and has been found to be involved in pathological processes such as cancer and development^44^. The experimental observations accumulated over many years suggest that somatic mutations are the typical genetic alterations that affect Ras. However, the mutation event for the RAS gene does not appear to be involved in the etiology of breast cancer in some patients^45^. We found that the KRAS gene was hypomethylated and was regulated by two miRNAs (hsa-miR-16 and hsa-miR-98). Moreover, these two miRNAs, which have been found to be implicated in the breast cancer development^46^, were aberrantly methylated. Therefore, the KRAS gene is strictly regulated by DNA methylation and miRNAs.

Taken together, our findings indicated that aberrant methylation of ncRNA regulators and target mRNAs, might result in the aberrant expression of genes and, subsequently, might cooperatively mediate pathway dysregulation, leading to the development and progression of breast cancer (Figure 7C).

## Discussion

In this study, we characterized genome-wide methylation patterns in breast cancer based on published high-throughput sequencing methylation data. Our study reveals that greater number of ncRNA promoters were aberrantly methylated, including lncRNAs and miRNAs. Moreover, we summarized five aberrant methylation patterns of ncRNA promoters and found that aberrant methylation not only on CGIs, but also on 5′ and 3′ CGI shores. Because of a lack of genome-wide methylation profiling, investigating the methylation patterns of CGI shores in breast cancer is difficult. Here, we found that the methylation patterns of both CGI shores in breast cancer are consistent with previous results for protein coding genes in colorectal cancer^47^ and prostate cancer^48^. Therefore, although coding RNAs and nc-RNAs function distinctly, they share similar aberrant methylation patterns.

Despite a growing consensus that long intergenic ncRNAs (lincRNAs) are modulators of cancer, the understanding of DNA methylation patterns of lincRNAs in cancer remains limited. In our study, lincRNAs were found to comprise the majority of the aberrantly methylated lncRNA promoters in the breast cancer samples. As intergenic CGIs are typically methylated in actively transcribed genes, it would be interesting to determine what proportion of these lincRNAs is located in an actively transcribed gene. H3K4me3 is a prominent histone mark associated with active genes, and we found that approximately 59.62% of the hypermethylated lincRNAs were occupied by H3K4me3 signals in HMECs. By contrast, only 13.11% of the hypomethylated lincRNAs were occupied by H3K4me3 signals in HMECs, indicating that these lincRNAs might be silenced in normal cells. Moreover, we found that most hypermethylated lncRNAs were associated with decrease H3K4me3 density, and that the hypomethylated lncRNAs were associated with elevated H3K4me3 density. These results indicate that DNA methylation and histone modifications are two mechanisms that reglate lncRNA expression.

Many studies have shown that the expression of ncRNAs can be modified by genetic variants, such as copy number variants (CNVs) and single nucleotide polymorphisms (SNPs). Here, we found that approximately 11.8% and 9.71% of the hypermethylated lncRNAs and miRNAs, respectively were located in regions of recurring deletions^49^. This result is consistent with recent observations that although individual genetic alterations appear to affect the gene expression and methylation levels, these effects are rare^50^. Our observations demonstrated that ncRNA expression regulated by not only genetic but also epigenetic factors, which is consistent with recent observations^51^,^52^. Next, we identified a panel of ncRNA biomarkers that effectively discriminated between cancer and control samples. Among the lncRNA biomarkers indentified in our study, 64.90% lncRNAs were also differentially expressed in the comparison of 84 breast cancer samples and 51 non-malignant breast tissue samples^53^. We found that the clustering of the expression of these lncRNAs also distinguished cancerous from noncancerous tissues (Figure S6). Finally, functional analysis indicated that aberrant methylation of ncRNAs widely disturbs processes associated with the development and progression of breast cancer.

Polycomb-targeted genes are frequent targets of aberrant DNA methylation in cancers, and some miRNA genes have been shown to be targeted by polycomb proteins. Therefore, we examined whether polycomb-targeted ncRNAs are more likely to be targets of aberrant DNA methylation in breast cancer. To this end, we used the repressed regions of a study by Ernst et al. as polycomb-targeted regions^54^. We found a significant overlap between the ncRNA promoters that were occupied by the polycomb and those that were aberrantly hypermethylated in cancer cells. Comparative analysis revealed that the ncRNA promoters that were targeted by polycomb were more likely to be hypermethylated in cancer. These observations provided additional evidence for the association between polycomb-mediated repression and aberrant DNA methylation in breast cancer. The high proportion of polycomb targets among ncRNA promoters may, to a certain extent, explain the high proportion of ncRNA promoters displaying aberrant methylation in cancer. However, it is possible that no direct functional association exists between these two phenomena. Such ncRNAs are unmethylated in normal cells, are repressed by polycomb proteins, and acquire DNA methylation as an alternative silencing mechanism. These ncRNAs, perform tumour-suppressive functions, are inducible in normal cells, and are repressed by polycomb. Hypothetically, the epigenetic switch to DNA methylation-mediated repression reduces the plasticity of the regulatory program of these ncRNAs, mandating the silencing of the key ncRNA regulators and contributing to the abnormal growth potential of the cell.

In summary, our data demonstrates the suitability of MBDCap-seq to investigate cancer methylomes and identifies novel epigenetically dysregulated ncRNAs (lncRNAs and miRNAs). This study presents the aberrant methylation patterns of ncRNAs, providing a highly valuable resource for the investigation of the epigenetic regulation of breast cancer.

## Methods

### Genome-wide DNA methylation profiles

To explore the DNA methylation pattern of ncRNAs in breast cancer, DNA methylation data generated using the MBDCap-seq protocol were obtained from CMS^23^, consisting of 77 cancer samples and 10 control samples. The genome-wide methylation intensity values were directly downloaded and quantified as the numbers of reads uniquely mapped to each 100-bp genomic bin. Next, the normalization was performed based on the linear method^55^.

### Genomic coordinates of ncRNA and protein-coding genes

We downloaded the human lncRNA annotation from GENCODE (version 18), and the primary miRNA genomic coordinates were obtained from the miRBase database (version 20.0)^12^. After converting the genomic location to hg18 using the LiftOver tool in the UCSC browser, 13,459 lncRNAs and 1866 pri-miRNAs were analyzed. In addition, the genomic coordinates of the protein-coding gene were downloaded from the UCSC browser, and the RefSeq table was used. Moreover, the ncRNA and mRNA promoters were defined as the +/−2 kb region surrounding the TSS of each lncRNA/mRNAs or the start site of each pri-miRNA^56^.

### Genome-wide expression of ncRNAs

The expression data assessed using RNA-seq for two breast cancer cell lines (MCF-7 and HCC1954) and one control cell line (HMEC) were considered, and the raw reads data were downloaded from the ENCODE project^57^ and the study by Hon et al.^1^. After mapping and aligning the RNA-seq data using the TopHat and Cuffdiff program according to the default parameters^58^, the expression levels of the lncRNAs were measured as ‘fragments per kilobase of exon model per million mapped reads’ (FPKM).

The miRNA expression profiles of the breast cancer tissue samples were downloaded from The Cancer Genome Atlas (TCGA) (Table S6), which were measured using miRNA-Seq. The reads per million (RPM) mapped to each miRNA were calculated, and a total of 1046 pre-miRNAs were analyzed.

### Annotation of genomic features

The coordinates of the CGIs were primarily obtained from the UCSC database. Similar to previous studies^47^, the 5′- and 3′- CGI shores were defined as the 2 kb on either side of each CGI.

### Identification of the differentially methylated ncRNAs

An ncRNA or protein-coding gene was considered to be differentially methylated only if at least one DMRs resided in or overlapped with its promoter region. Here, we required that the overlap region include at least 50% of the DMR. Furthermore, we detected both the CGI and CGI shore regions displaying differential methylation by using the same procedure.

To detect the DMRs, differentially methylated bins were first identified. A 100-bp genomic bin was considered to display significantly altered methylation levels between breast cancer and control samples when following two criteria were met: (i) 2-fold or greater change in the methylation level and (ii) adjusted P < 0.01, which was calculated using Wilcox rank-sum test followed by multiple testing correction using the BH method. Furthemore, consecutive hyper-/hypo- methylated bins with no gap were merged into DMRs.

### Identification of the patterns of aberrant methylation

The aberrant ncRNA methylation patterns were further investigated in the context of CGIs, and five methylation patterns were classified: (1) aberrant methylation predominatly confined to the CGIs, in which the overlap region within the promoter was greater than 50% of either region; (2) aberrant methylation was strictly located 5′ of the CGIs; (3) aberrant methylation was strictly located 3′ of the CGIs; (4) aberrant methylation that overlapped with the CGI shores or an overlap of less than 50% between the CGIs and the DMR; and (5) aberrantly methylated promoters lacking CGIs.

### Aberrant expression of ncRNAs regulated by DNA methylation

To investigate the change in expression of each ncRNA caused by its aberrant methylation, differential expression analysis was performed. The differentially expressed miRNAs were detected using student's t-test followed by multiple testing correction using the BH method (adjusted p-values at 5% thresholds). The lncRNAs displaying greater than two-fold changes in expression in the cell lines were considered to be differentially expressed. Considering the repressive effects of DNA methylation, we further required opposing directional changes between methylation and expression.

### Validation of the ncRNA biomarkers

The quality of the lncRNA or miRNA biomarkers was evaluated using plotting ROC curves at various thresholds of the Z-score-transformed methylation or expression levels. First, we performed Z-score transformation on the methylation or expression levels across the samples for each lncRNA or miRNA. Then, we summarized the Z-scores for these ncRNAs into one score for each sample. The validation procedure was conducted using the MedCalc program.

## Supplementary Material

Supplementary Information Supplementary files

Supplementary Information Table S1

Supplementary Information Table S3

Supplementary Information Table S4

Supplementary Information Table S5
