# Supplementary material for: Genome-wide DNA methylome analysis reveals epigenetically dysregulated non-coding RNAs in human breast cancer
Source: Sci Rep. 2015 Mar 5;5:8790. doi: 10.1038/srep08790 (PMC4350105; doi:10.1038/srep08790)
Supplement: Supplementary Information — Supplementary files [file srep08790-s1.docx]

**Genome-wide DNA methylome analysis reveals epigenetically dysregulated non-coding RNAs in human breast cancer**

Yongsheng Li^1,3^, Yunpeng Zhang^1,3^, Shengli Li^1,3^, Jianping Lu^1^, Juan Chen^1^, Yuan Wang^1^, Yixue Li^1,2,^*, Juan Xu^1,^*, Xia Li^1,^*

**Affiliations:**

^1^College of Bioinformatics Science and Technology, Harbin Medical University, Harbin, China.

^2^ShanghaiCenter for Bioinformation Technology, Shanghai, People’s Republic of China.

^3^These authors contributed equally to this work

***Correspondence**:

Pro. Xia Li, College of Bioinformatics Science and Technology, Harbin Medical University, Harbin 150081, China (Email: lixia@hrbmu.edu.cn). Phone: 86-451-86615922; Fax: 86-451-86615922

Pro. Juan Xu, College of Bioinformatics Science and Technology, Harbin Medical University, Harbin 150081, China. Email: [xujuanbiocc@ems.hrbmu.edu.cn](mailto:xujuanbiocc@ems.hrbmu.edu.cn)

Pro. Yixue Li, ShanghaiCenter for Bioinformation Technology, Shanghai, People’s Republic of China.Email: [yxli@sibs.ac.cn](mailto:yxli@sibs.ac.cn)


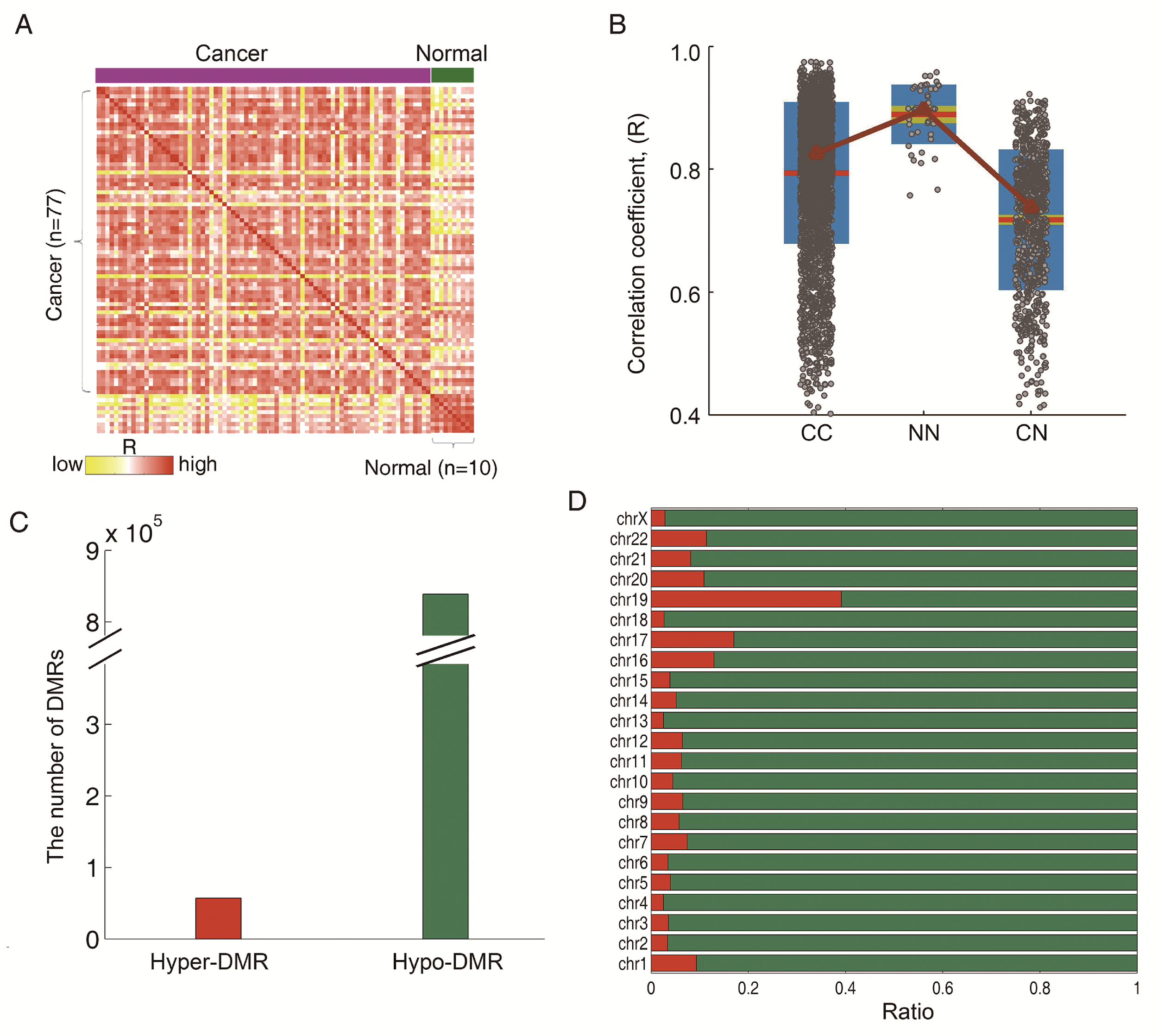


**Figure S1. Global DNA methylation profiles highlight clear difference in breast cancers and normal controls.** (A) DNA methylation was calculated from MBD-seq data using 1kb bins across the genome, and the relationship among samples was determined by Pearson correlation. (B) The distribution of Pearson correlation among samples. Red lines indicate the mean correlation coefficient and the light green areas represent of the 95% confidence interval of mean. The dark blue areas indicate the stand error of the correlation coefficient, and the median correlation coefficient is fitted by the dark red line. Each dot represents the correlation between two samples. (C) The number of DMRs identified in the comparison between breast cancer and controls. (D) Stacking barplots showing percentage of hyper and hypomethylated DMRs out of all bins for each chromosome.


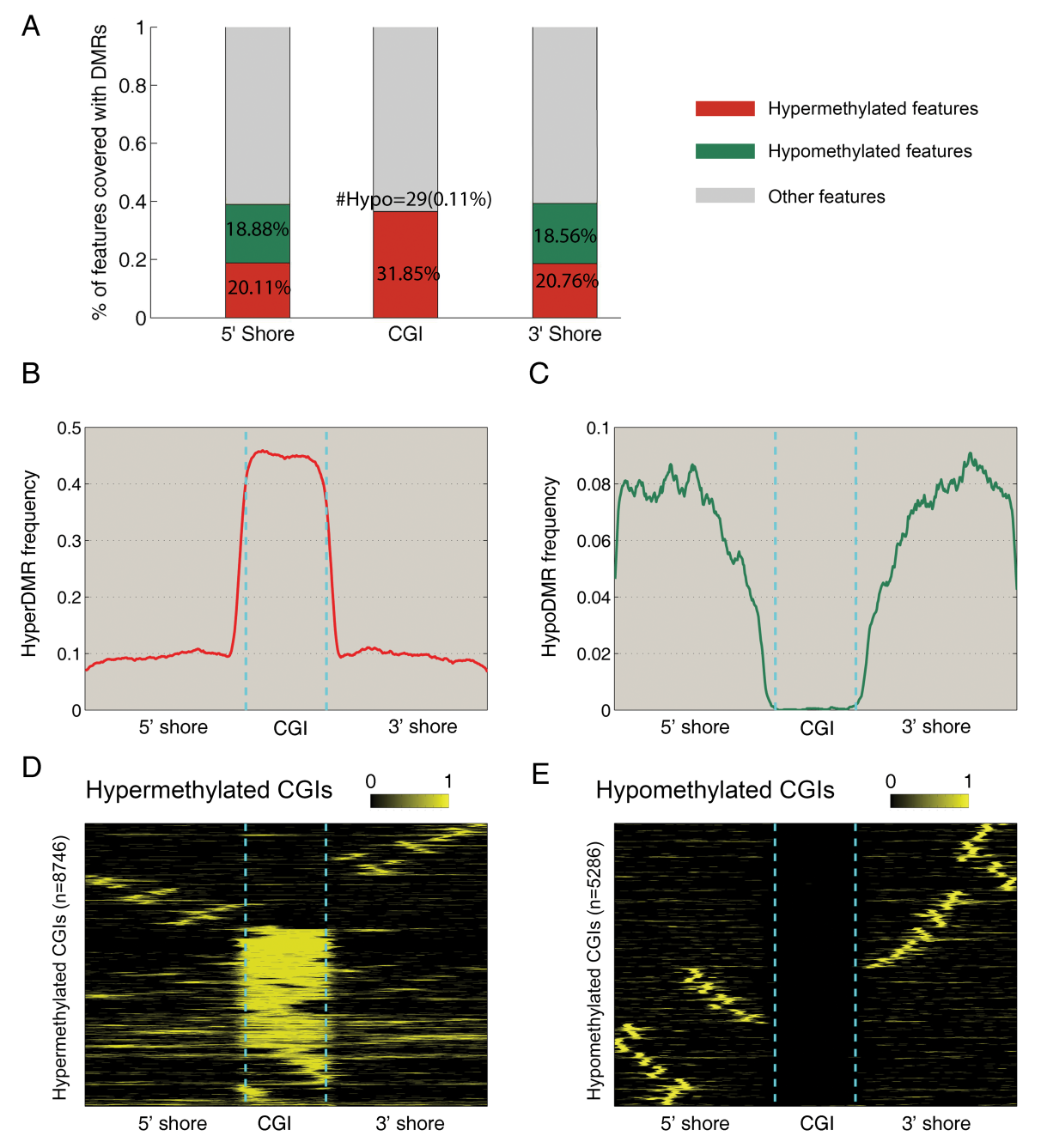


**Figure S2. Extensively CGI hypermethylation in breast cancers.** (A) Stacking barplots representing the percentage of hyper- and hypomethylated CGIs and shores. (B) and (C) show the average aberrant hyper- and hypo methylation frequency around the CGIs, respectively. show (D) and (E) the aberrant hyper- and hypomethylation around the CGIs. Each row represents a unique CGI and corresponding shores, which was divided into 500 windows. The aberrant methylation frequency is indicated in yellow.


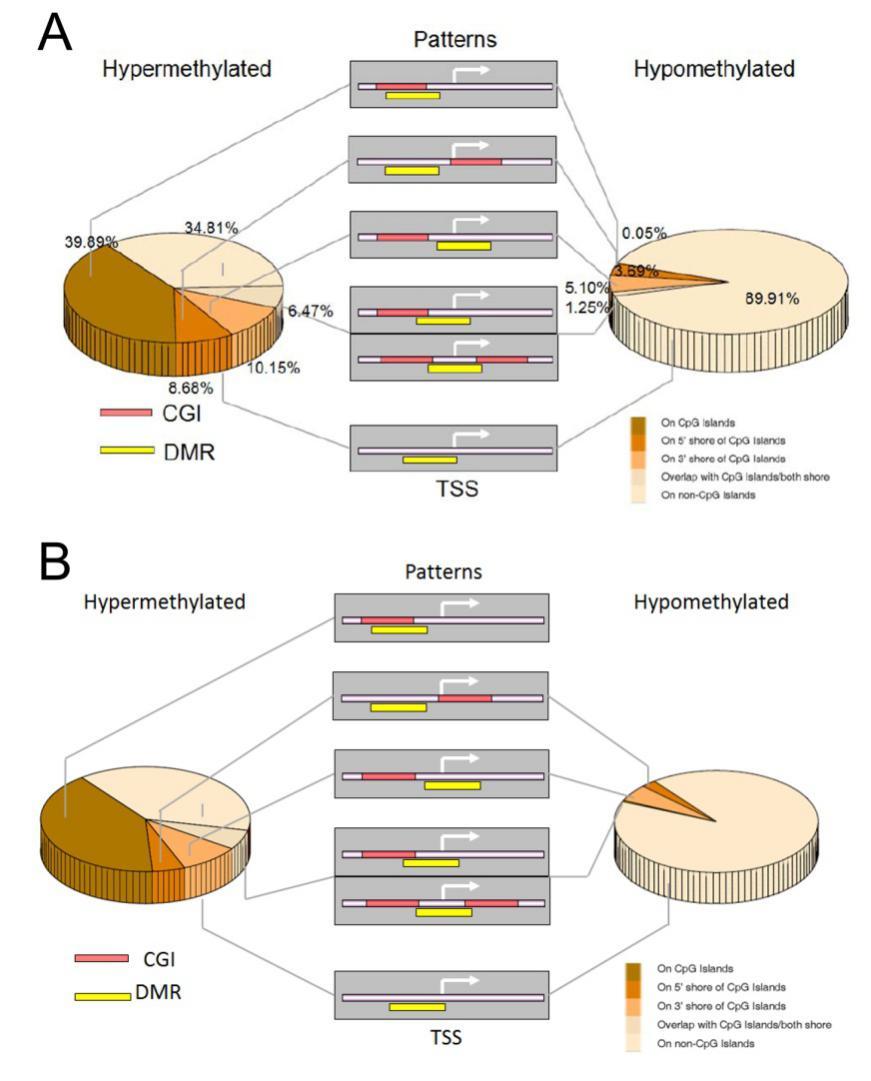


**Figure S3. The proportion of distinct patterns of lncRNA and miRNA promoters aberrant methylation.** The middle-panel is schematic diagram for distinct methylation patterns, while the left and the right is the ratio of distinct methylation patterns for hyper- or hypo-methylated promoters. (A) lncRNA promoters, (B) miRNA promoters.

**
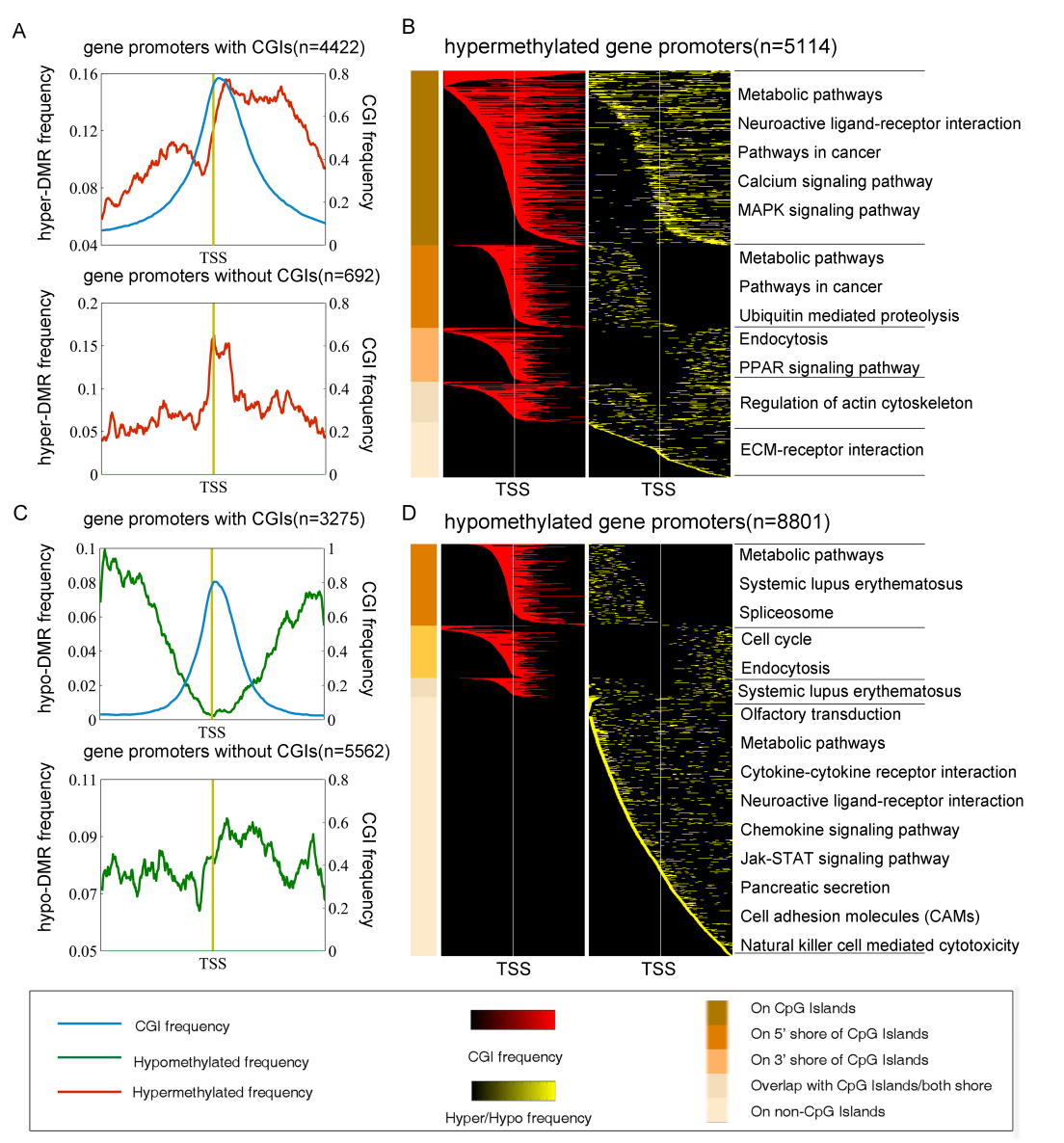
**

**Figure S4. Aberrant methylation patterns around the TSSs of protein coding genes in the breast cancers.** (A) Average aberrant hypermethylation frequency of 4422 promoters with CGIs. And average aberrant hypermethylation frequency of 692 promoters that lacked of CGIs. (B) Heat map of CGI frequency and aberrant hypermethylation frequency in breast cancer. A total of 5114 gene promoters that harbored hypermethylation (yellow) were shown. Each row represents a unique promoter region at 10-bp window size, covering +2kb flanking the transcription start sites. The location of a CGI (red) in the aberrant methylated gene promoters is shown in the first column. Promoters are ordered by the location of methylation on a CGI, adjacent to the island (shore) or promoters that lacked CGI as represented with different shades of brown on the left. Enriched pathways and example genes in each group are shown to the right. (C) Average aberrant hypomethylation frequency of 3275 promoters with CGIs. And average aberrant hypomethylation frequency of 5562 promoters that lacked of CGIs. (D) Heat map of CGI frequency and aberrant hypomethylation frequency in breast cancer.


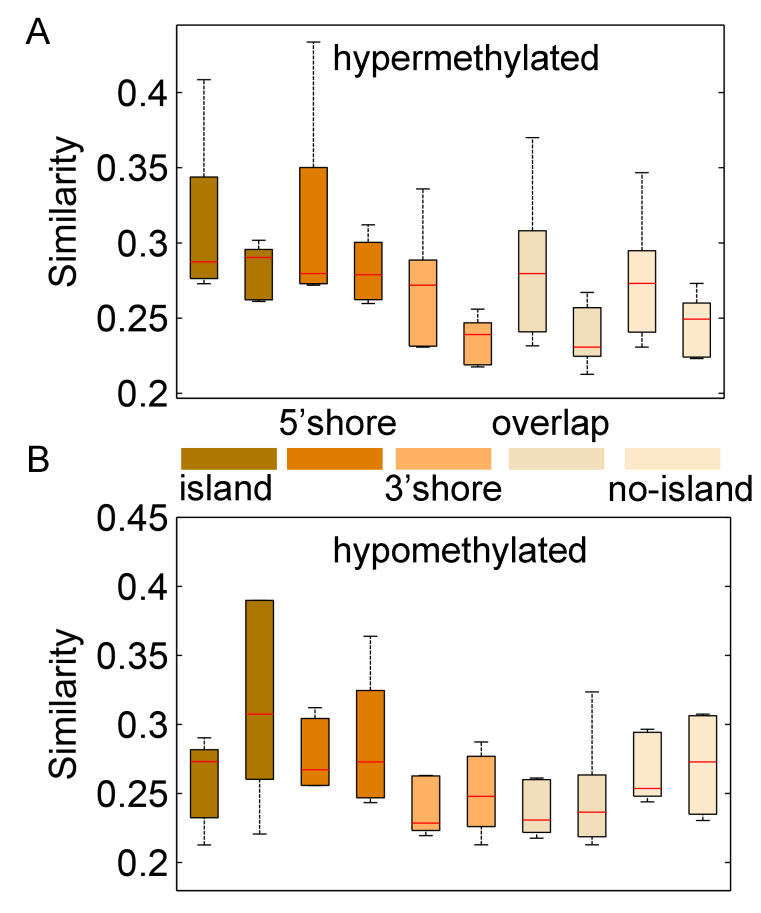


**Figure S5. Genes that have the methylation change in the same direction were with high similarities.** (A) Hypermethylated genes were similar with hyper-genes than hypo-genes. For each pattern, the first box shows the similarities between hyper-genes and the second box shows the similarities between hyper- and hypo-genes. (B) Hypomethylated genes were similar with hypo-genes than hyper-genes. For each pattern, the first box shows the similarities between hyper- and hypo-genes and the second box shows the similarities between hypo-genes.


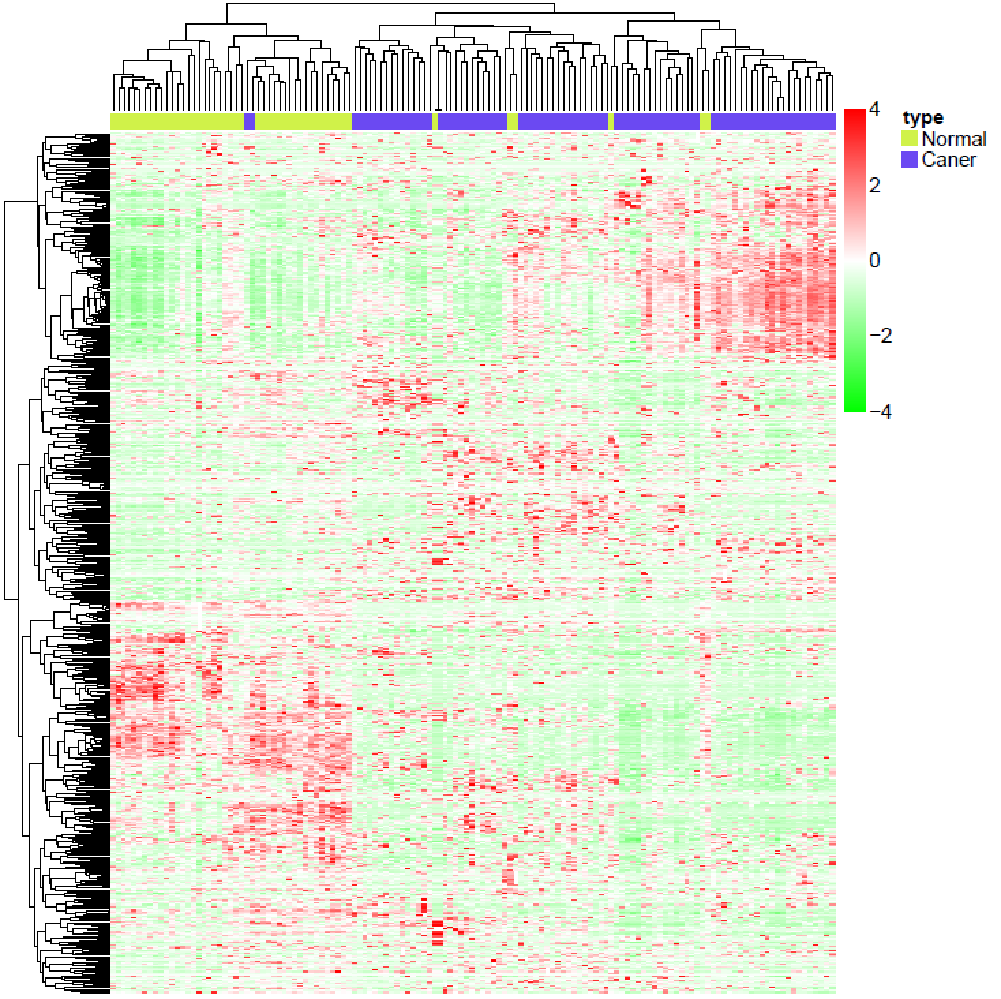


**Figure S6. Hierarchical clustering of lncRNA biomarkers included in the expression profiles of clinal samples.** 84 breast cancer samples and the 51 paired adjacent non-tumor samples were analyzed. Some cancer samples were profiled repeatly.

**Supplemental Tables**

**Table S1. Aberrant methylated ncRNA promoters in breast cancer.**

**Table S2. The number of the aberrantly methylated promoters for each distinct aberrant methylation pattern.**

| **CGIs type** | **Pattern** | **Promoters** | **Number**  **(hyper)** | **Ratio**  **(hyper)** | **Number**  **(hypo)** | **Ratio**  **(hypo)** |
| --- | --- | --- | --- | --- | --- | --- |
| **With**  **CGIs** | **On CGIs** | **lncRNA** | 542 | 39.89% | 3 | 0.05% |
|  |  | **miRNA** | 113 | 40.65% | 19 | 2.37% |
|  |  | **Gene** | 2201 | 43.04% | 10 | 0.11% |
|  | **On 5’-shore** | **lncRNA** | 118 | 8.68% | 234 | 3.69% |
|  |  | **miRNA** | 14 | 5.04% | 43 | 5.36% |
|  |  | **Gene** | 1036 | 20.26% | 1741 | 19.78% |
|  | **On 3’-shore** | **lncRNA** | 138 | 10.15% | 323 | 5.10% |
|  |  | **miRNA** | 26 | 9.35% | 0 | 0% |
|  |  | **Gene** | 675 | 13.20% | 1115 | 12.67% |
|  | **Others with CGI** | **lncRNA** | 88 | 6.47% | 79 | 1.25% |
|  |  | **miRNA** | 17 | 6.12% | 3 | 0.37% |
|  |  | **Gene** | 510 | 9.97% | 409 | 4.65% |
| **Without**  **CGIs** | **Without CGIs** | **lncRNA** | 473 | 34.81% | 5697 | 89.91% |
|  |  | **miRNA** | 108 | 38.84% | 737 | 91.90% |
|  |  | **Gene** | 692 | 43.04% | 5526 | 62.79% |

**Table S3. The ncRNA biomarkers in breast cancer.**

**Table S4. The KEGG pathways regulated by the lncRNA biomarkers.**

**Table S5. The KEGG pathways regulated by the miRNA biomarkers.**

**Table S6. The DNA methylation and gene expression profiles used in the study.**

| **Type** | **Dataset** | **Platform** | **Samples** | **Source** |
| --- | --- | --- | --- | --- |
| Methylation | CMS | MBDCap-seq | 77 cancer/10 normal | CMS |
| MiRNA-exp | TCGA | miRNA-Seq | 21 cancer/19 normal | TCGA |
| lncRNA exp | ENCODE, GEO | RNA-seq | HMEC, MCF-7, HCC1954 | ENCODE, GEO(GSE29069) |
| H3K4me3 | ENCODE, GEO | ChIP-seq | HMEC, MCF-7, HCC1954 | ENCODE, GEO(GSE29069) |
| H3K27me3 | ENCODE, GEO | ChIP-seq | HMEC, MCF-7, HCC1954 | ENCODE, GEO(GSE29069) |

CMS: Cancer Methylome System; TCGA: The Cancer Genome Atlas

**Supplemental materials and methods**

**Text S1**

**Histone modification of ncRNAs in breast cancers**

ChIP-seq data (H3K4me3 and H3K27me3) for HMEC and MCF-7 was downloaded from ENCODE and the ChIP-seq data for HCC1954 cell lines were obtained from NCBI Gene Expression Omnibus (GEO) database (GSE29069)[^1^](#_heading=h.1t3h5sf). The reads were mapped to human genome (hg18) by the software Bowtie[^2^](#_heading=h.1t3h5sf), and only reads that at most three mismatches were retained for subsequential analysis. The ChIPDiff program[^3^](#_heading=h.4d34og8) with default parameters was used for quantitative comparison of the histone modification levels in the breast cancer cells and the normal cell lines. The genomic 1kb bins were first identified as differential histone modification sites (DHMS) and consecutive DHMSs with no gap between them were merged into DHMS regions.

**Function enrichment analysis**

KEGG pathways and gene ontology (GO) analysis were performed to find enriched pathways using the WebGestalt[^4^](#_heading=h.2s8eyo1). P-values were multiple tests corrected in order to reduce false-positive rates. Pathways or GO terms with adjusted p-values of <0.01 and with at least two interesting genes were considered significant.

**Gene set functional similarity**

The growing availability of genome-scale datasets has attracted increasing attention to the development of computational methods for automated inference of functional similarities among gene sets. In order to explore if the genes with similar aberrant methylation patterns have higher functional similarities, we used the GS2 (GO-based similarity of gene sets) to measure the gene set similarity[^5^](#_heading=h.17dp8vu). The measure quantifies the similarity of the GO annotations among a set of genes by averaging the contribution of each gene’s GO term and their ancestor terms with respect to the GO vocabulary graph. We downloaded the Python code (<http://bioserver.cs.rice.edu/gs>2) and computed the functional similarities among gene sets.

**Collection of miRNA targets**

MiRNA is a small non-coding RNA molecule found in plants and animals, which functions in transcriptional and post-transcriptional regulation of gene expression. The function of miRNAs appears to be through their target genes. Currently, several online databases that predict binding sites and target genes of miRNAs are available, such as PicTar, TargetScan, and miRanda. Among these algorithms, TargetScan has demonstrated the best performance compared to others[^6^](#_heading=h.3rdcrjn). Therefore, we extracted the miRNA-gene pairs from the TargetScan server (version 6.2)[^7^](#_heading=h.26in1rg). We further required that the miRNA family were conserved. In addition, the manually curated experimentally validated miRNA-gene interactions were kindly provided by the authors of TarBase[^8^](#_heading=h.lnxbz9).

**Clustering analyses**

To explore if the miRNA or gene biomarkers discovered in the CMS dataset can be used as diagnosis markers, hierarchical clustering of the methylation or expression was done using the function of ‘clustergram’ in Matlab with average linkage. In the analysis of methylation profiles, we used the Spearman correlation to perform the hierarchical clustering while using the Euclidean distance in the expression profiles.

**Collection of known ncRNAs associated with breast cancers**

The breast cancer associated miRNAs were collected from three databases, miR2Disease[^9^](#_heading=h.35nkun2) and HMDD[^10^](#_heading=h.1ksv4uv). All of these two databases aim at providing a comprehensive resource of miRNA deregulation in various human diseases. And the associations of miRNA and disease were manually retrieved from literatures. We searched these databases and retrieved the association between the 26 miRNAs and breast cancer. As a result, we found that most of the 26 miRNAs identified in our study were reported to be associated with breast cancer. In addtion, we review the evidence linking lncRNAs to diverse human diseases in pubmed. And the lncRNAs that were associated with diseases supported by literture were marked in the supplemental tables.

***References***

1 Hon, G. C. *et al.* Global DNA hypomethylation coupled to repressive chromatin domain formation and gene silencing in breast cancer. *Genome research* **22**, 246-258, doi:10.1101/gr.125872.111 (2012).

2 Langmead, B. & Salzberg, S. L. Fast gapped-read alignment with Bowtie 2. *Nat Methods* **9**, 357-359, doi:10.1038/nmeth.1923 (2012).

3 Xu, H., Wei, C. L., Lin, F. & Sung, W. K. An HMM approach to genome-wide identification of differential histone modification sites from ChIP-seq data. *Bioinformatics* **24**, 2344-2349, doi:10.1093/bioinformatics/btn402 (2008).

4 Zhang, B., Kirov, S. & Snoddy, J. WebGestalt: an integrated system for exploring gene sets in various biological contexts. *Nucleic acids research* **33**, W741-748, doi:10.1093/nar/gki475 (2005).

5 Ruths, T., Ruths, D. & Nakhleh, L. GS2: an efficiently computable measure of GO-based similarity of gene sets. *Bioinformatics* **25**, 1178-1184, doi:10.1093/bioinformatics/btp128 (2009).

6 Selbach, M. *et al.* Widespread changes in protein synthesis induced by microRNAs. *Nature* **455**, 58-63, doi:10.1038/nature07228 (2008).

7 Lewis, B. P., Burge, C. B. & Bartel, D. P. Conserved seed pairing, often flanked by adenosines, indicates that thousands of human genes are microRNA targets. *Cell* **120**, 15-20, doi:10.1016/j.cell.2004.12.035 (2005).

8 Vergoulis, T. *et al.* TarBase 6.0: capturing the exponential growth of miRNA targets with experimental support. *Nucleic Acids Res* **40**, D222-229, doi:10.1093/nar/gkr1161 (2012).

9 Jiang, Q. *et al.* miR2Disease: a manually curated database for microRNA deregulation in human disease. *Nucleic Acids Res* **37**, D98-104, doi:10.1093/nar/gkn714 (2009).

10 Li, Y. *et al.* HMDD v2.0: a database for experimentally supported human microRNA and disease associations. *Nucleic acids research*, doi:10.1093/nar/gkt1023 (2013).
